# Esophageal Melanocytosis in Oral Opium Consumption

**DOI:** 10.5812/ircmj.7820

**Published:** 2014-01-05

**Authors:** Bita Geramizadeh, Fatemeh Asadian, Alireza Taghavi

**Affiliations:** 1 Department of Pathology, Shiraz University of Medical Sciences, Shiraz, IR Iran; 2 Transplant Research Center, Shiraz University of Medical Sciences, Shiraz, IR Iran; 3 Department of Internal Medicine, Gastroenterology Research Center, Shiraz University of Medical Sciences, Shiraz, IR Iran

**Keywords:** Melanosis, Opium, Mouth

## Abstract

Esophageal melanocytosis is a rare and benign condition, characterized by melanocytic proliferation of the esophageal squamous epithelium with heavy melanin deposition. The etiology and pathogenesis has not been exactly known but it seems to be a chronic stimulus such as gastroesophageal reflux. This condition is very rare and about 35 cases have been reported so far, most of which have been from India and Japan. Herein, we present a case of esophageal melanocytosis in a patient with long history of oral opium consumption. To the best of our knowledge, such a history has not been reported.

## 1. Introduction

Esophageal Melanosis or melanocytosis is a rare and benign condition, which is first described by De La Pava in 1963 (1). After that, about 35 cases have been reported in the English literature most of which were from India (2). No definite pathogenesis has been proposed for esophageal melanocytosis; however, it seems to develop secondary to a chronic stimulation such as long term gastroesophageal reflux (2). Herein, we report our experience in an old man with a long history of oral opium poppy consumption, who developed abdominal pain and dysphagia and turned out to have esophageal melanocytosis. To the best of our knowledge, this association has not been reported before.

## 2. Case Report

A 75 year-old man was referred to gastroenterology clinic with the chief complaint of epigastric pain and dysphagia. His past medical history was unremarkable. No positive family history was identified. He has been a heavy smoker since 40 years ago. He has also been an opium addict since 30 years ago. He used to consume opium poppy until 10 years ago, but since then he has been using opium orally. The entire basic laboratory tests results including white blood cell count, hemoglobin, and renal and liver function tests were within normal limit.

**Figure 1.**
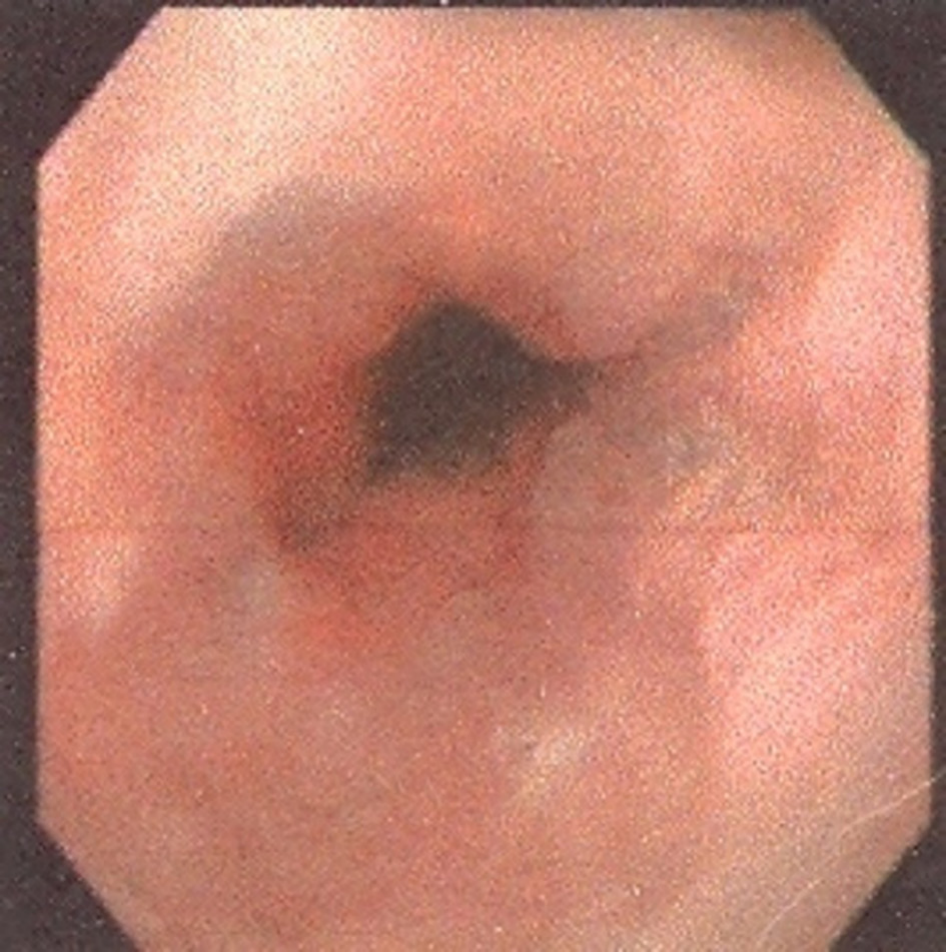
Upper Endoscopic View of the Middle Esophagus Shows Pigmented Lesion of the Mucosa

**Figure 2.**
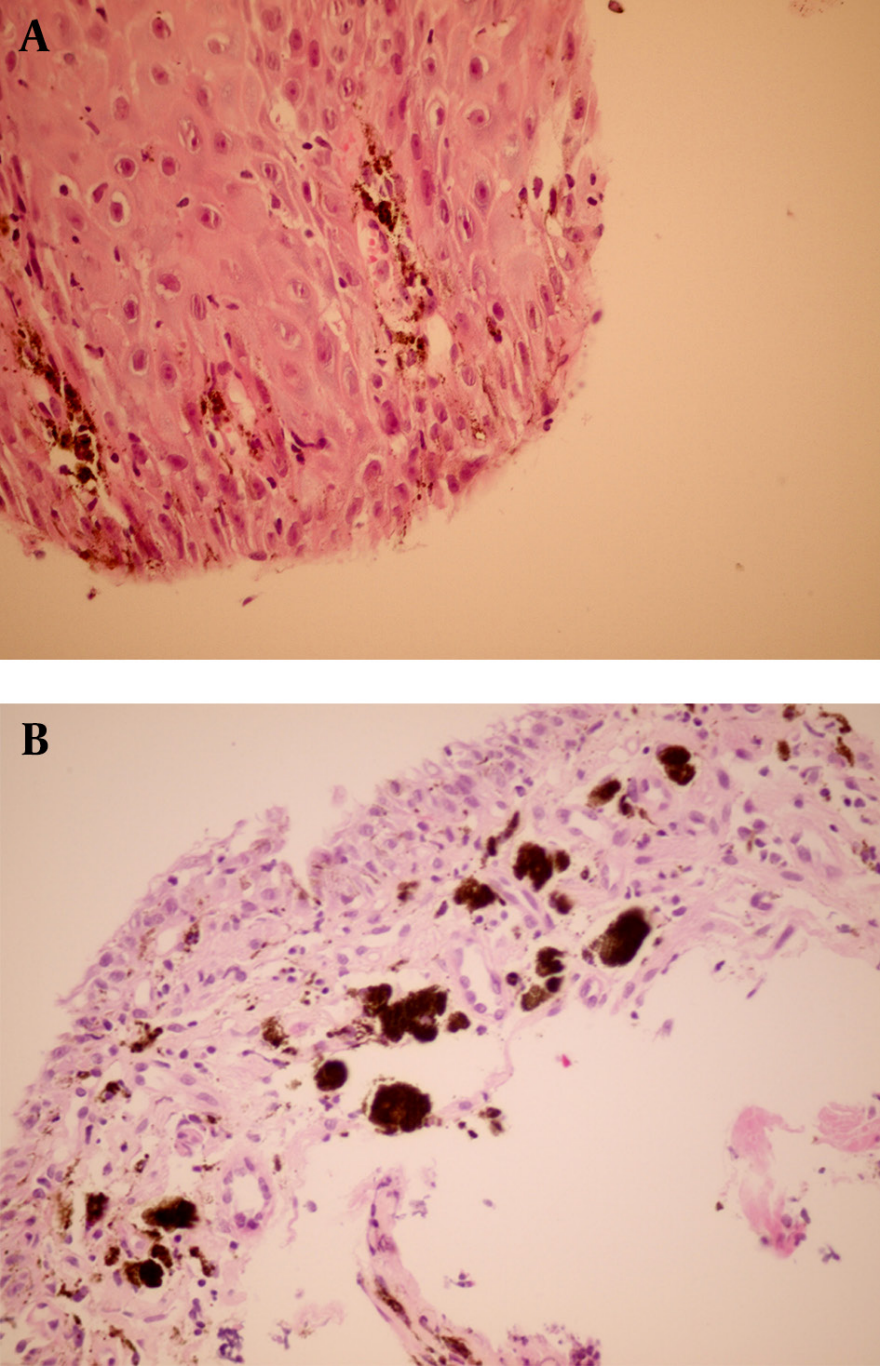
A. Microscopic Section From the Surface Squamous Epithelium Shows Scattered Pigmented Cells Between the Keratinocytes. (H & E X400). B. Lamina Properia Shows Many Heavily Pigmented Cells. (H & E X250)

**Figure 3.**
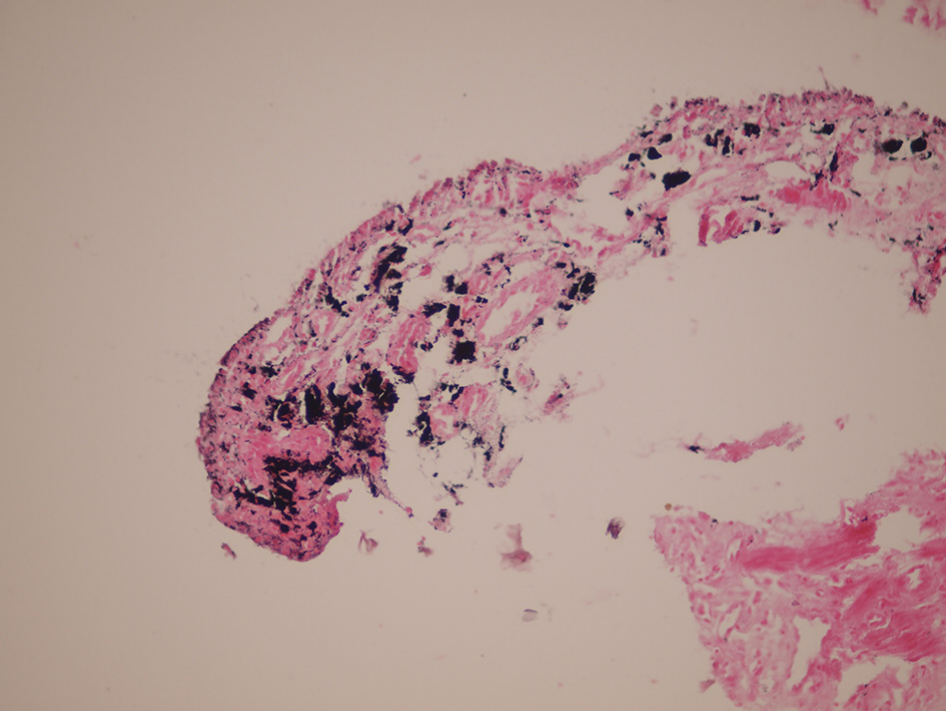
The Above Mentioned Cells Positive by Masson Fontana Stain. (Masson Fontana X250)

Results of physical examination of the heart, lungs, abdomen and anogenital region were completely normal. Findings of computed tomography (CT) scan of chest and abdomen were normal. Conventional upper endoscopy showed black discoloration in the middle part of the esophagus (Figure 1). Stomach and duodenum findings were normal. Colonoscopy findings were also within normal limit. Multiple biopsies were taken from the black spot. Hematoxylin and Eosin (H & E) sections showed increased numbers of pigment-laden dendritic melanocytes and deposition of coarse brownish black pigment within the basal layer of the squamous epithelium and also in the lamina properia (Figure 2 A, B). These cells were positive by Masson Fontana (Figure 3). There was no cellular atypia. The cells were negative in Perls’ stain. HMB 45 and S100 were also positive. The overlying squamous epithelium was normal except for the presence of the above-mentioned pigment laden cells. Supportive therapy was started for gastroesophageal reflux. Although he refused follow up endoscopy, follow up was unremarkable after 6 months.

## 3. Discussion

Esophageal melanocytosis is a rare and benign disease defined as melanocytic proliferation in the basal epithelium of esophagus with increased quantity of the melanin in the mucosa (3). It is recommended to use the term melanocytosis instead of melanosis to describe the lesion, because the melanocytic proliferation is better emphasized by the term melanocytosis (3). Epidemiologically, this disease is very rare in western countries and less than five cases have been reported so far from west and most of the previously reported cases have been from India and Japan (4, 5).

The etiology and pathogenesis remain uncertain; however it may be the result of gastroesophageal reflux and other continued and chronic stimuli (6). It has also been reported in Addison’s disease, Laugier-Hunziger syndrome, oral melanoma, esophageal squamous cell carcinoma, and in about 30% of patients with esophageal melanoma (2). Our case is a unique one in regard to the etiology that seems to be the chronic stimulation of oral opium consumption. The main challenging aspect of this disease is explaining the reason for the melanocytes presence in the esophagus. During embryogenesis, melanocytes begin to migrate from neural crest to other parts of the body such as meninges, uvea, and oral cavity. Therefore, it can be hypothesized that these cells can also reach several different locations in the body such as esophagus. Another theory is the differentiation of multipotential stem cells in the basal layer of esophageal epithelium. In addition, any chronic stimulus that causes keratinocyte hyperplasia, such as heavy alcohol consumption, is able to increase the number of melanocytes (4-7).

The presenting symptoms in the majority of the previous cases have been dysphagia and abdominal discomfort (3). Upper endoscopic finding in the previous reports has been described as flat and oval lesions with irregular borders and black granular spots (6). Histologic studies in the patients with isolated esophageal melanocytosis have shown hyperpigmentation and melanocytic proliferation without atypia in the surface epithelial and lamina properia (7). Our patient presented with abdominal pain and dysphagia. Upper endoscopy showed black pigmentation in the middle segment of esophagus. Histologic examination revealed many pigment laden cells both between the keratinocytes and in the lamina properia. These pigments were negative with perls’ stain for iron and positive with Masson Fontana stain, and the stain was wiped out after bleaching techniques. These techniques can exclude other pigment lesions such as hemosiderosis, anthracosis, and exogenous dye ingestion (6).

Other differential diagnoses in the histology and endoscopy are blue nevus and malignant melanoma (3). There is no histologic atypia in esophageal melanosis in contrast to esophageal malignant melanoma, which reveals atypical spindle to epithelioid cells (6). Blue nevus is an extremely rare condition in the esophagus, which can be differentiated from esophageal melanocytosis by the presence of the clusters of heavily pigmented dendritic cells in the stroma with no junctional activity (3). Esophageal melanocytosis is benign and there is no need for treatment or surveillance (2). Finally, although esophageal melanocytosis is uncommon and many gastroenterologists and pathologists lack experience with this entity, our case emphasizes that it can be seen, especially in the patients with the history of oral consumption of different stimuli such as opium.
